# Controlled exposure to particulate matter from urban street air is associated with decreased vasodilation and heart rate variability in overweight and older adults

**DOI:** 10.1186/s12989-015-0081-9

**Published:** 2015-03-19

**Authors:** Jette G Hemmingsen, Jenny Rissler, Jens Lykkesfeldt, Gerd Sallsten, Jesper Kristiansen, Peter Møller P, Steffen Loft

**Affiliations:** Department of Public Health, Section of Environmental Health, Faculty of Health and Medical Sciences, University of Copenhagen, Øster Farimagsgade 5A, DK-1014 Copenhagen K, Denmark; Division of Ergonomics and Aerosol Technology, Department of Design Sciences, Lund University, P.O. Box 118, SE-221 00 Lund, Sweden; Department of Veterinary Disease Biology, Faculty of Health and Medical Sciences, University of Copenhagen, Ridebanevej 9, 1870 Frb. C. Copenhagen, Denmark; Department of Occupational and Environmental Medicine, Sahlgrenska University Hospital and Academy, Gothenburg, Sweden; The National Research Centre for the Working Environment, Lersø Parkalle 105, 2100 Copenhagen, Denmark

**Keywords:** Traffic emission, Particulate matter, Cardiovascular disease, Endothelial function, Heart rate variability, Oxidative stress, Ascorbic acid, Tetrahydrobiopterin, Elderly, Obesity

## Abstract

**Background:**

Exposure to particulate matter (PM) is generally associated with elevated risk of cardiovascular morbidity and mortality. Elderly and obese subjects may be particularly susceptible, although short-term effects are poorly described.

**Methods:**

Sixty healthy subjects (25 males, 35 females, age 55 to 83 years, body mass index > 25 kg/m^2^) were included in a cross-over study with 5 hours of exposure to particle- or sham-filtered air from a busy street using an exposure-chamber. The sham- versus particle-filtered air had average particle number concentrations of ~23.000 versus ~1800/cm^3^ and PM_2.5_ levels of 24 versus 3*μ*g/m^3^, respectively. The PM contained similar fractions of elemental and black carbon (~20-25%) in both exposure scenarios. Reactive hyperemia and nitroglycerin-induced vasodilation in finger arteries and heart rate variability (HRV) measured within 1 h after exposure were primary outcomes. Potential explanatory mechanistic variables included markers of oxidative stress (ascorbate/dehydroascorbate, nitric oxide-production cofactor tetrahydrobiopterin and its oxidation product dihydrobiopterin) and inflammation markers (C-reactive protein and leukocyte differential counts).

**Results:**

Nitroglycerin-induced vasodilation was reduced by 12% [95% confidence interval: −22%; −1.0%] following PM exposure, whereas hyperemia-induced vasodilation was reduced by 5% [95% confidence interval: −11.6%; 1.6%]. Moreover, HRV measurements showed that the high and low frequency domains were significantly decreased and increased, respectively. Redox and inflammatory status did not change significantly based on the above measures.

**Conclusions:**

This study indicates that exposure to real-life levels of PM from urban street air impairs the vasomotor function and HRV in overweight middle-aged and elderly adults, although this could not be explained by changes in inflammation, oxidative stress or nitric oxide-cofactors.

**Electronic supplementary material:**

The online version of this article (doi:10.1186/s12989-015-0081-9) contains supplementary material, which is available to authorized users.

## Background

Exposure to particulate matter (PM) particularly in terms PM_2.5_ (mass of PM with aerodynamic diameter < 2.5 μm) in ambient air is associated with elevated risk of cardiovascular morbidity and mortality especially in risk groups with old age, obesity and/or diabetes [1,2]. The physiological mechanisms that link PM exposure and cardiovascular disease are not fully understood, but oxidative stress and inflammation are thought to be important with three plausible biological pathways leading to vasomotor dysfunction, arrhythmia, atherosclerotic progression, thrombogenesis and plaque instability: i) deposited PM trigger pulmonary inflammation with release of inflammatory and vasoactive mediators into the systemic circulation, ii) deposited ultrafine particles (aerodynamic diameter <100 nm) may translocate directly from the lungs to the bloodstream causing oxidative stress and inflammation with direct effects on the endothelium, or iii) deposited PM may activate neuronal signaling from the lungs with indirect effects on the cardiovascular function [1,3].

Exposure to ambient air PM has been associated with vasomotor dysfunction in both animal models and in humans [4]. Normal vessels are protected from shear stress hemodynamic forces by vasodilation dependent on activation of endothelial nitric oxide synthase (eNOS) that generates NO by converting L-arginine to L-citrulline in the presence of molecular oxygen. Tetrahydrobiopterin (BH_4_) is an essential cofactor for eNOS. The oxidized form of BH_4_, dihydrobiopterin (BH_2_), can cause eNOS uncoupling with production of superoxide anion radicals, which react with NO forming peroxynitrite, a potent oxidant [5,6]. An animal study indicated that exposure to diesel exhaust, the most important source of urban ultrafine particles, increased vasoconstriction through decreased NO bioavailability by uncoupling eNOS and decreased levels of BH_4_ [7]. *Ex vivo* exposure of animal aorta segments to diesel exhaust PM directly inhibited the relaxation response, whereas this response was restored by addition of superoxide dismutase that degrades superoxide anion radicals [8]. Moreover, studies with controlled exposure to diesel exhaust in humans have indicated that vasodilation response was impaired both related to endothelial stimulation to NO production and to administration of an NO donor [3].

Ascorbic acid (AA) is a potent intracellular and circulatory antioxidant, which together with its 2-electron oxidation product, dehydroascorbate (DHA), are used as biomarkers for oxidative stress in plasma [9]. Moreover, AA appears to increase the NO bioavailability and alleviate endothelial dysfunction in patients with cardiovascular disease [10]. This is possibly through preventing oxidation of BH_4_, promoting recycling of BH_4_ from its oxidized form, and/or through increasing gene transcription or activity of eNOS [10]. In addition, AA is important for maintenance of the endothelial barrier function and regulation of NADPH oxidase activity involved in the inflammatory response [11].

Heart rate variability (HRV) is a measure of changed cardiac autonomic function, which has been linked to risk for cardiovascular morbidity and mortality [12]. HRV has been widely studied in relation to exposure to ambient and occupational air pollution as recently summarized in a meta-analysis of 29 studies from 1999–2011 [13]. This supported an inverse relationship between exposure to PM and especially time domains of HRV expressed as standard deviation of NN intervals (SD_NN_) as well as changes in high frequency (HF) and low frequency (LF) domains. Acute exposure in traffic has been associated with a decline in HRV [14], whereas no change in HRV was seen after 1-h exposure to a high concentration of diesel exhaust [15]. Elderly humans may be particularly susceptible to effects of ambient air PM on HRV [16].

The aim of this randomized cross-over study was to assess the effect of 5-h exposure to PM from urban street air on endothelium dependent and independent vasomotor function and HRV time and frequency domains in a cohort of overweight middle-aged and elderly subjects believed to be more sensitive to the biological and physiological responses to inhaled urban air PM, although not yet documented consistently with respect to clinical outcomes [17]. Potential mechanisms of adverse effects were studied in terms of oxidative stress assessed as AA and DHA, BH_4_ availability, and inflammation markers in terms of leukocyte differential counts.

## Results

### Exposure levels

**Figure 1:**
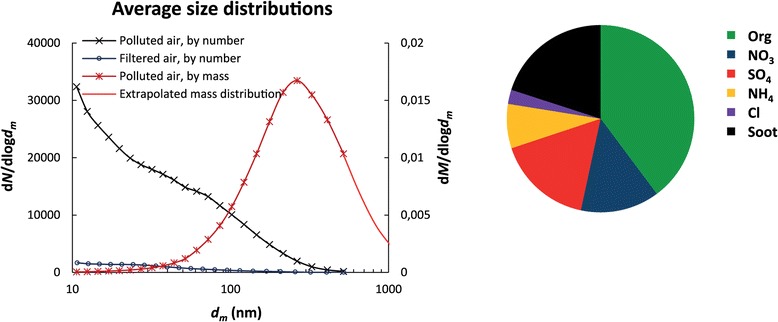
**Average particle number size distribution and relative composition of particulate matter with diameter <1 μm in the exposure chamber assessed by a Scanning Mobility Particle Sizer, a Differential Mobility Analyzer coupled in series with an Aerosol Particle Mass Analyzer and an Aerosol Mass Spectrometer.**

The main average exposure levels are given in Table 1. Participants were exposed to non-filtered air with average particle concentrations of 24 μg/m^3^ (PM_2.5_) and ~23, 000/cm^3^ with size distribution depicted in Figure 1. From the number size distributions and the effective densities the average PM_1_ was estimated to ~11-12 μg/m^3^. The particle number concentration in the exposure chamber was nearly 1:1 with the levels outdoor, compensating for different cut-offs of the two condensation particle counters used indoor and outdoor, respectively. This indicated that the transmission of particles from outdoor was high. Potentially, some of the volatile fraction of the particles might get lost during temperature conditioning of the air which was needed during winter time to keep the temperature in the exposure chamber stable. Filtering the air removed ~90% of the particles and slightly reduced concentration of NO_2_ (Table 1). Key drivers of the limited variation in air pollutants would be the day-to-day variation in wind direction, air mass origin and traffic intensity.

**Table 1:** **Characterization of exposure concentrations in the chamber with and without filtration of the inlet air from an urban street**

	**Non-filtered air**	**Particle-filtered air**
Particle number concentration, CPC (number/cm^3^)	23174 ± 6857	1779 ± 773
PM_2.5_ continuously by Dusttrak (μg/m^3^)	24 ± 13	3.0 ± 1.2
PM_2.5_ filter based (μg/m^3^)	18 ± 3	1.5 ± 1.5
Black carbon from PM_2.5_ filters (μg/m^3^)	3.9 ± 0.9	0.3 ± 0.3
Sum of PAH filter based (ng/m^3^)	2.5 ± 1.1	0.025 ± 0.025
NO (μg/m^3^)	31 ± 19	33 ± 14
NO_2_ (μg/m^3^)	45 ± 13	26 ± 26
NO_x_ (μg/m^3^)	77 ± 31	59 ± 38

Values are mean ± SD, where SD corresponds to the day-to-day variation.

Approximately 50% of the number of particles found at street level corresponds to fresh soot particles emitted from local traffic estimated from DMA-APM data [18] covering a size range of 50 to 450 nm. By mass, the corresponding fraction is only 20-25% explained by the low effective density of the aggregates at the peak in the submicron mass size distributions (e.g. ~250 nm).

The chemical composition [19] of the non-refractory PM_1_ mass was measured by means of aerosol mass spectrometry omitting the soot mass [18]. By combining with the soot mass fraction measured using a Differential Mobility Analyzer coupled in series with an Aerosol Particle Mass Analyzer (DMA-APM), the total chemical composition was estimated (Figure 1). It should be noted that the detailed characterisation was performed only during the first half of the exposures, and that the composition varied with the history of the air mass (long range transport), as discussed elsewhere [18].

### Vasomotor function

**Figure 2:**
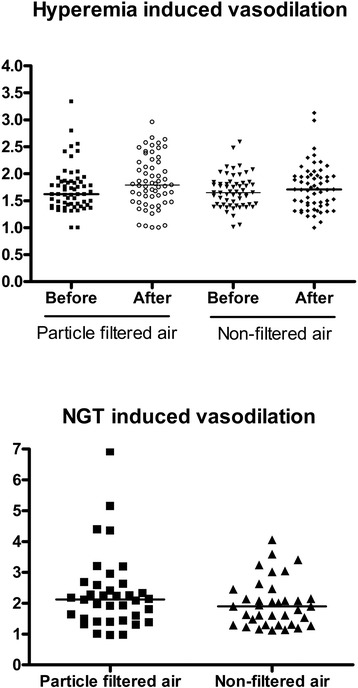
**Individual and median vasodilation induced by reactive hyperemia (n = 60) before and after and nitroglycerin (NTG) (n = 40) only after exposure to particle filtered air versus non-filtered air from an urban street in 60 middle-aged and elderly overweight subjects.**

Vasodilation was measured as induced by reactive hyperemia (RHI: reactive hyperemia index) and an NO donor (NTG-I: nitroglycerin-induced vasodilation index), representing endothelium dependent and independent mechanisms, respectively (Figure 2). RHI was 5% (95% CI: −11.6; 1.6) lower after 5-h exposure to PM as compared with the response after filtered air although not being statistically significant (P = 0.128). However, the NTG-I was statistically significantly reduced by 12% (95% CI: −22; −1.0) after the exposure to PM from urban street air (P = 0.033), although only 40 participants had this measurement due to a history of possible migraine or limited availability of medical supervision.

### Heart rate variability

After 5-h exposure to PM from urban street air HRV was significantly altered in the frequency domains with a significant reduction of 6.0% (95% CI:-11; −1.1) in HFn and a significant increase of 7.5% (95% CI: 0.8; 15) in LFn, whereas there was no statistically significant difference in the time domain in terms of SD_NN_ irrespective of baseline adjustment (Table 2). However, the SD_NN_ was significantly reduced by 13% (95% CI:-24; −0.3; p = 0.045) immediately after entering the exposure chamber with non-filtered after as compared with the parallel measurement immediately after entering the chamber with filtered air.

**Table 2:** **Heart Rate Variability in subjects at the beginning of and after 5-h exposure in a chamber with or without filtration of inlet air from an urban street**

**Exposure**	**Particle filtered air**		**Non-filtered air**	
	**After entry**	**After 5 h**	**After entry**	**After 5 h**
SD_NN_, ms	68 (41;115)	48 (27;82)	60* (31;116)	52 (26;99)
HFn	28 (16;55)	31 (10;58)	30 (11;69)	29 (13;54)*
LFn	68 (27;89)	66 (30;90)	66 (27;90)	70 (37;86)#
LF/HF ratio	2.4 (0.6;5)	2.1 (0.5;10)	2.3 (0.4;8)	2.5 (0.8;7)

*p < 0.05 (decrease), #p = 0.027 (increase), SD_NN_: standard deviation of the beat-to-beat interval; HFn: High frequency, normalised units; LFn: Low frequency, normalized units. Values are medians (5th;95th percentiles).

### Blood pressure, lipid profile, and metabolic markers

Median values of systolic and diastolic blood pressure, lipids (cholesterol and triglycerides), and metabolic biomarkers (fasting glucose and Hb_A1c_) were within the normal reference ranges and confirmed the health status of the participants (Additional file 1: Table S1). The values were not affected by the exposure to non-filtered street air.

### Oxidative stress and inflammation markers

The oxidative stress biomarkers BH_4_, BH_2_, biopterin, uric acid, AA and DHA changed over time (before and after air exposure), but there were no differences between exposure to PM from street air or filtered air (Table 3). Inflammation biomarkers including C-reactive protein (CRP) and white blood cell (WBC) counts (leukocytes, neutrophils, lymphocytes, monocytes, and eosinophils) showed no exposure-related changes.

**Table 3:** **Oxidative stress and inflammation biomarkers in subjects before and after 5-h exposure in a chamber with or without filtration of inlet air from a street**

	**Particle filtered air**		**Non-filtered air**	
	**Before**	**After**	**Before**	**After**
BH_4_ (mmol/L)	11.7 (7.7;16.8)	9.5 (5;17.3)	12.1(6.9;18.4)	10.6 (4.5;15.6)
BH_2_ + biopterin (mmol/L)	6.2 (4.2; 9)	4.9 (3.6;7.9)	6.5 (4.4;8.6)	4.6 (3.1;6.6)
Total biopterins (mmol/L)	18.1 (13.9;24)	14.6 (10.6;24.6)	19 (13.9;25)	15.5 (9.9;20.1)
BH_2_/BH_4_ (mmol/L)	0.52 (0.31;0.96)	0.46 (0.3;1.13)	0.53 (0.34;0.83)	0.44 (0.27;1.20)
Uric acid (mmol/L)	304 (152;421)	298 (157;440)	290 (163;449)	314 (171;431)
Ascorbic Acid (mmol/L)	56 (14;88)	57 (6;91)	56 (10;90)	61 (14;90)
Dehydroascorbic acid (mmol/L)	1.16 (0;5.0)	1.2 (0;4.4)	1 (0;5.0)	1.2 (0;5.5)
%Dehydroascorbic acid	2.4 (0;14.6)	2.5 (0;9.9)	2.1 (0;11.1)	1.7 (0;9.4)
C-reactive protein (mg/L)	1.1 (0.3;4.5)	1.1 (0.3;5.5)	1.1 (0.35;6.1)	1.1 (0.3;6.3)
Leukocytes (×10^9^ cells/L)	5.2 (2.9;8)	5.85 (3.7;9.6)	5.2 (3.3;8)	6.2 (4.3;10.2)
Neutrophils (×10^9^ cells/L)	2.7 (1.7;4.2)	3.25 (1.9;5.2)	2.9 (1.4;4.6)	3.5 (2.1;5.4)
Lymfocytes (×10^9^ cells/L)	1.8 (0.7;3.1)	1.95 (0.9;3.5)	1.8 (0.9;3.1)	2.05 (0.9;3)
Monocytes (×10^9^ cells/L)	0.2 (0.1;0.5)	0.3 (0.1;0.4)	0.2 (0.1;0.4)	0.3 (0.2,0.5)
Eosinophils (×10^9^ cells/L)	0.2 (0.1;0.5)	0.1 (0.1;0.5)	0.2 (0.1;0.4)	0.2 (0.1;0.5)

BH_4_ tetrahydrobiopterin; BH_2_ Dihydrobiopterin. Values are medians (5th;95th percentiles).

## Discussion

In this study, we found that 5-h exposure to PM from urban street air was associated with a statistically significant 12% reduction in NGT-I vasodilation and a 5% non-significant decrease in hyperemia-induced vasodilation in a possibly sensitive cohort of middle-aged and elderly overweight subjects. In addition, the HRV measurements indicated significantly decreased HFn and increased LFn after the 5-h exposure, whereas SD_NN_ was decreased immediately after entering the chamber with PM. However, these vascular and cardiac effects were not associated with altered explanatory biomarker levels of oxidative stress, cofactors of NO production or inflammation.

Controlled exposure studies have demonstrated that 1-h inhalation of diesel exhaust at high concentrations (300 μg/m^3^) with exercise leads to impaired vasomotor function in young healthy subjects and cardiac ischemia in patients with coronary heart disease [20-22]. Here, we show that even at PM concentrations encountered at the street level (PM_2.5_ and black carbon concentrations of 24 and 3.9 μg/m^3^, respectively), vasomotor function was impaired in 55 to 83 years old overweight subjects after 5-h exposure without exercise. In support, ambient particle number concentrations were recently associated with low RHI among middle-aged subjects in Copenhagen, Denmark [23].

NO is a key endothelium-dependent vasodilating mediator and several lines of evidence have suggested that reduced NO bioavailability is associated with impaired vasomotor function after PM exposure [3,24]. A recent study using animal models suggested that diesel exhaust exposure, possibly through oxidative stress, depleted BH_4_ with formation of BH_2_ leading to uncoupling of eNOS and generation of superoxide and peroxinitrite at the expense of NO [7]. A possible role of BH_4_ is supported by the observation that co-infusion of BH_4_ (500 mg/min) can restore the reduced endothelium-dependent forearm vasodilation elicited by administration of acetylcholine in hypersensitive patients [25]. However, we found no change in BH_4_ or BH_2_ levels in relation to the PM exposure. Moreover, we observed primarily an altered endothelium-independent vasodilation, elicited by administration of NTG. Similarly, controlled exposure studies with pure diesel exhaust have found reduced responses to NO-related vasodilation elicited endothelium-dependently by bradykinin or acetylcholine and endothelium–independently by sodium nitroprusside [3,8]. The use of an NO synthase inhibitor (L-NMMA) in such a study indicated that NO generation is actually enhanced, but NO consumption is much further increased with resulting reduced bioavailability [3].

The reduced NO bioavailability due to consumption by ROS after airway exposure is supported by animal studies involving engineered nano-TiO_2_ [26]. Similarly, superoxide production appeared to explain impaired vasomotor function in rat aorta segments exposed to diesel exhaust particles *ex vivo* [8]. AA is an important antioxidant in both lung lining fluid [27] and plasma where it can increase NO bioavailability and restore endothelial function possibly by scavenging superoxide, preventing oxidation of BH_4_ and increase the expression and activity of eNOS [10]. Moreover, reduced levels of AA and high percentage of the oxidation product DHA in plasma are biomarkers of oxidative stress as shown in active and passive smokers [9,11]. Preincubation of human umbilical vein endothelial cells with AA for 24 hours reduced oxidative stress induced by 3-h exposure to exhaust PM from a light duty diesel engine supporting a role of AA for vascular effects of PM [28]. However, we found no effect of the exposure on the plasma levels of AA or DHA. This is consistent with lack of effect on AA following 2-h exposure to diesel exhaust at 200 μg/m^3^ in 10 adults with metabolic syndrome [29]. That study also found no effect on the lipid peroxidation products (F2-isoprostanes) and 8-oxo-7,8-deoxy-2’-guanosine in urine, measured by ELISA methods. Nevertheless, elevated levels of these biomarkers have been consistently associated with exposure to ambient air pollution [30]. Similarly, exposure to street air in Copenhagen, Denmark or in Cotonou, Benin, was associated with increased oxidative stress-induced DNA damage in peripheral blood mononuclear cells [31-33]. Although, the present exposure to PM from street air or diesel exhaust did not affect the levels of AA, DHA, BH_4_ or BH_2_ to an extent that explains the changed vasomotor function likely due to increased consumption of NO, it cannot be excluded that AA and BH_4_ concentrations were changed locally in the vessel wall without being reflected in forearm venous plasma.

Decreased HRV is recognized as a risk factor for cardiovascular morbidity and mortality [34] and a recent meta-analysis concluded that decreased HRV in both time and frequency domains are associated with high ambient air PM levels [13]. In contrast, a controlled exposure study with diesel exhaust (300 μg/m^3^) for one hour with 32 healthy subjects and 20 patients with prior myocardial infarction showed no change in any of the HRV domains [15]. However, the same research group found that 3 hours exposure to wood smoke at similar mass level was associated with decreased HRV time and HF domains in 14 young healthy subjects, whereas the LF domain was marginally increased although not statistically significant [35]. In partial agreement with those results we found a significant decrease in the HF domain and an increase in the LF domain after exposure. This is also consistent with a decrease in HF HRV in an older group of research volunteers after controlled 2-h exposure to concentrated fine particulate matter from an airshed dominated by local traffic sources, long-range traffic-related PM and coal-burning power plants [16]. Controlled 2-h exposure to concentrated ambient air coarse PM in Los Angeles, USA, appeared to have similar effects with decreased parasympathetic influence on HRV in both asthmatic and healthy volunteers [36]. Moreover, there was a decrease in the HF domain in subjects with type 2 diabetes exposed to elemental carbon ultrafine particles at 50 μg/m^3^ for 2 hours [37], whereas a similar setup with healthy subjects showed no such HRV change [38]. In young female subjects, the HF domain was increased and the LF domain unchanged after exposure to burning candle emissions at PM concentrations of 200 μg/m^3^ for 30 and 90 min [39]. Accordingly, exposure for more than two hours might be required for detection of adverse HRV changes, where the HF domain could be most sensitive for 5 min recordings and elderly, obese and/or type 2 diabetics may be most susceptible. Although we after the 5-h exposure found no sign of change in the time domain of HRV irrespectively of baseline adjustment, SD_NN_ was significantly reduced just after entry into the chamber with non-filtered air. This suggests an immediate, possibly neurally-mediated, effect and it also points to a weakness in our design where the measurements of HRV at baseline and at the end of exposure had to be performed under the same conditions. Another weakness with respect to the time domain of HRV is the short sampling time of 5 min.

Short-term high level exposure to urban air pollution (mean PM_2.5_ levels of 147μg/m^3^) and ozone (mean 121 ppb) have previously been shown to increase blood pressure [40], whereas no such effect was found after controlled exposure to diesel exhaust at even higher concentrations [15]. At our level of exposure to urban air PM and low ozone concentration there was no effect on blood pressure.

Elevated WBC counts and CRP are signs of inflammations. Controlled 1-h exposure to diesel engine exhaust did not affect WBC counts [3], whereas similar 3-h exposure levels appeared to increase monocyte and total leukocyte counts 20 hours later [41]. Similarly, 2-h exposure to concentrated ambient air PM with intermittent exercise caused an increase in pulmonary neutrophils, but no change in total leukocyte counts [42]. Elevated CRP levels and risk of diabetes have mainly been associated with long-term exposure to air pollution [43,44]. In our study WBC counts, CRP and metabolic markers including lipid profile, glucose levels and Hb_A1C_ were all in normal range despite the participants’ overweight and showed no change after 5-h PM exposure.

Our study had a strong cross-over design and a high statistical power with 60 overweight middle-aged and older adults, with supposed particular susceptibility, serving as their own control. However, possibly due to their weight-status our participants showed more day-to-day variation in RHI than we previously found among elderly subjects and we did not have power to show significance of a 5% decrease, although we could show a significant decrease in NTG-I among the 40 participants with this measurement. Moreover, the study of real-life exposure led to some variation in both the levels and composition for each exposure session. We could not control exposure leading up to the study sessions except to avoid PM exposure by having the participants use a face mask on their way from home to the exposure chamber. It was only possible to perform physiological measurements and blood sampling on the participants before or in the beginning and just after the 5-h exposure. Thus, we could not assess the time course of effects during exposure and late effects might have been missed.

## Conclusions

PM exposure from traffic caused vasomotor dysfunction and reduced HRV in overweight middle-aged and elderly subjects indicating adverse cardiovascular effects at real-life exposure levels. The vasomotor dysfunction appeared related to depletion of NO rather than reduced endothelial production of NO.

## Materials and methods

### Study population

Participants were invited by posting notices in local newspapers and handing out flyers in the area of Copenhagen, Denmark. We recruited 60 healthy, middle-aged and older (>55 years), overweight (body mass index, BMI > 25 kg/m^2^), nonsmoking (defined as cessation of smoking at least 1 year before the study) participants (25 men and 35 women) with no personal history of cardiovascular diseases (see Additional file 1: Table S2 for further characteristics). Of the participants 27 were never-smokers, whereas the 33 former smokers stopped smoking on average 20 years prior to the study. Subjects taking vasoactive drugs were not allowed into the study, but some participants took medicine in terms of analgesic or antirheumatic drugs (11), hormone related drugs (10), antihistamines (5), sedatives or antidepressants (5), lipid lowering drugs (3), proton pump inhibitors (2), bronchodilators (2), antibiotics (2) or none (28). The participants were asked to maintain their usual life-style, diet and use of medicine throughout the study period. The study was reviewed and approved by The Committees on Health Research Ethics in the Capital Region of Denmark (H-3-2011-074) and in accordance with the Helsinki II declaration. All participants were given both oral and written information and provided written consent before the study.

### Study design

This study design was cross-over, repeated measures, where participants served as their own control with a blinded and randomized order of exposure to particle filtered or non-filtered outdoor air. Each participant was studied twice with 5 hours in an exposure chamber and around 14 days between exposures, which included 1–4 participants simultaneously. The participants were instructed to wear a highly efficient face mask (Dust Respirator 8812; 3M, St. Paul, MN, USA) on the way from their home to the exposure chamber in order to prevent exposure to ambient air PM immediately before the experiments. This mask type has been shown to reduce symptoms and improve cardiovascular health measures in patients with cardiovascular disease walking in polluted air in Beijing, China [20].

The participants arrived fasting except for possible morning medicine and were served a standard continental breakfast after all baseline measurements were done. During the 5-h exposure, they were at rest and only allowed to leave the chamber in order to go to the bathroom. We collected blood samples and measured blood pressure and vasomotor function before entry into the exposure chamber and within 1 hour after exposure. All measurements were completed within a 7-month period, from November 2011 to end of May 2012.

To create exposure scenarios simulating real-life exposure in traffic, air from the curb-side of a street (Østersøgade) in central Copenhagen, Denmark, was introduced directly into the exposure chamber (collected ~5 m from the nearest tailpipe area). The traffic density on this road was 26,800 vehicles during daytime (6 am to 6 pm), of which 2.2% were diesel powered heavy duty (>3.5 tons) and 35% of the light duty vehicles have diesel-powered engines. The outdoor air was continuously pumped into the exposure chamber using two KVR-100 Channel ventilators (Øland A/S, Ballerup, Denmark) at 230 m^3^/h (pressure = 100 Pa) resulting in an air exchange of 5.3/h in the chamber. Heating devices, placed within the airstream, kept the chamber temperature constant. To create either high or low PM exposure levels in the chamber, the outdoor air was passed through custom built units, with or without High Efficiency Particulate Adsorption filters (Camfil FARR HEPA filter 226002A1; Camfil A/S Stockholm, Sweden). In both scenarios (with or without filtration), air flow and pressure were constant, whereas nitrogen oxides (NO, NO_2_), ozone and carbon monoxide were minimally affected by the filtration.

### Exposure assessment

The air in the exposure chamber was monitored continuously throughout all exposures with respect to particle number concentrations and PM_2.5_ by means of a TSI 3007 condensation particle counter and a Dusttrak Aerosol Monitor 8520 equipped with a PM_2.5_ inlet (TSI, St. Paul, MN, USA), respectively. On most exposure occasions, the particle number size distributions was monitored using a custom built Scanning Mobility Particle Sizer, covering particles of diameters in the range 10–600 nm [18], whereas NO and NO_2_ were monitored using a chemiluminescence NOx monitor (Thermo 42i monitor, Theromo Scientific). On 16 exposure days with sham-filtration and 8 days with active filtration, PM_2.5_ from the exposure chamber was collected on Teflon filters (Pall Teflo 47mm) for mass and black carbon analysis by means of a PQ100 Basel PM_2.5_ sampler (EPA WINS) (BGI Inc., Waltham, MA, USA). The polycyclic aromatic hydrocarbon content on the filters was measured using gas chromatography–mass spectrometry (GC-MS) [45].

During the first half of the study, an intense campaign characterizing the outdoor air, at the precise same location as the exposure chamber study, was performed. Instruments included were a Differential Mobility Analyzer coupled in series with an Aerosol Particle Mass Analyzer [46] (DMA-APM, model 3600, Kanomax, Japan), a high-resolution time-of-flight aerosol mass spectrometer [19] (HR-ToF-AMS, Aerodyne Research Inc., USA) and a ultrafine condensation particle counter (UCPC, TSI model 3025) running continuously outside the chamber allowing an estimation of the difference in concentrations at the curb-side and inside the exposure chamber. The DMA-APM allows size resolved determination of the particle effective density, which was used when determining the particle mass size distributions and total mass concentrations from the number size distributions. By extrapolating the mass-size distributions up to 1 *μ*m using a log-normal distribution function, we showed that PM_0.6_ typically made up ~80-90% of PM_1_. The DMA-APM can differentiate between fresh soot aggregates from more compact aged particles when externally mixed [18]. The high-resolution time-of-flight aerosol mass spectrometer allows online determination of the chemical composition of the non-refractory particulate mass.

### Vasomotor function

Vasomotor function was measured non-invasively using the EndoPAT2000 (Itamar Medical Ltd, Cesaria, Israel) as described further in the Additional file 1 [32,47,48]. RHI was recorded in a finger probe as the vasodilation response to hyperemia after 5 min occlusion of the brachial artery flow with reference to a finger probe on the contralateral arm. After each exposure scenario, we subsequently measured the vasodilation induced in the contralateral arm by 5 mg nitroglycerin (NTG), an NO donor, placed under the tongue 5 min after the RHI measurement. NTG was administered to only 40 subjects, whereas a possible history of migraine or limited capacity for medical supervision precluded this treatment in the remaining subjects. NTG-I was calculated as the ratio of average amplitudes of the pulse wave signal after and before administration.

### Heart rate variability

HRV was measured by Actiheart (CamNtech Ltd, U.K.) placed on the chest before entering the exposure chamber (see Additional file 1 for details). Analysis of HRV was done for the 5 min just after the participants had entered the chamber and the 5 min just before leaving. For each period we calculated the standard deviation (SD_NN_) of the interbeat intervals and the power for the LF (range 0.04-0.15 Hz) and HF (range 0.15-0.4 Hz), expressed in normalized units as HFn and LFn by dividing with the power in the frequency range 0.04-0.5 Hz, respectively.

### Biomarkers in blood

In a droplet (20 μl) of blood, we determined glycated hemoglobin levels, white blood cell (WBC) count and differential profile: lymphocytes, monocytes, eosinophils and neutrophils with a multiplatform analyzer (Chempaq XBC, Denmark). Plasma CRP, total cholesterol, high-density lipoprotein, low-density lipoprotein and triglycerides were analyzed at the Department of Clinical Biochemistry, Copenhagen University Hospital.

Both AA and BH_4_ are highly susceptible to oxidation and well validated methods for immediate handling and preservation of blood samples and for analyses were applied [49,50]. For determination of plasma AA and DHA 4 ml of venous blood was drawn into EDTA tubes and immediately placed on ice to avoid further oxidation. Plasma was within one min separated by centrifugation (16000g, 3 min, 4°C) and 400 μl was added to a MPA solution (10% meta-phosphoric acid w/v, 2 mM Na_2_EDTA). After centrifugation of this (16000g, 1 min, 4°C) the supernatant was stored at −80°C until analysis for AA and DHA by HPLC with electrochemical detection as described previously [51].

For determination of plasma BH_2_ and BH_4_, blood was drawn into K_3_-EDTA tubes containing 100 μl of a freshly made solution of dithioerythritol prepared by dissolving 40 mg in 1000 μl milli-Q water. After gentle turning the plasma was separated within one min by centrifugation (3000g, 3 min, 4°C) and stored at −80°C until analysis. BH_2,_ BH_4_ and biopterin were determined by HPLC as previously described [49].

#### Statistical analysis

All statistical analyses were performed using Stata/IC software (version 13.0). Linear mixed effect models (*xtmixed*) were used to evaluate the effect of PM exposure on log-transformed outcomes for a normal distribution of the residuals. All outcomes in terms of measurements after exposure to non-filtered or filtered street air were adjusted for baseline level (before entering the exposure chamber) in order to account for day-to-day variation within an individual. For NTG-I no baseline value was available. Adjustment for BMI, age and gender were included to account for missing values for a few subjects. The percentage change in outcomes related to statistically significant effects of PM exposure was calculated with 95% confidence intervals from exponential transformation of the regression coefficients. Statistical significance was taken at P < 0.05. The sample size was based on the intra-individual variation in RHI found among elderly where an 8% change was found after filtration of the home indoor air for 48 h without using baseline adjustment [32]. A similar effect size would require 37 participants with type I and II error levels of 5% and 10%, respectively. A total of 60 participants were recruited in order to have sufficient power for adjustment for baseline values measured in the morning before exposure.

## Additional file

Additional file 1:
**Measurement of vasomotor function.** Measurement of Heart rate variability (HRV). **Table S1.** Blood pressure and metabolic biomarkers in subjects before and after 5-h exposure in a chamber with or without filtration of inlet air from an urban street. **Table S2.** Characteristics of the study participants as number or median (5;95% percentiles).

## Abbreviations

AA: Ascorbic acid
BH_2_: Dihydrobiopterin
BH_4_: Tetrahydrobiopterin
BMI: Body mass index
DHA: Dehydroascobate
DMA-APM: Differential mobility - aerosol particle mass analyzer
eNOS: Endothelial nitric oxide synthase
HF: High frequency
HRV: Heart rate variability
LF: Low frequency
NTG: Nitroglycerin
NGT-I: Nitroglycerin-induced vasodilation index
NO: nitric oxide
PM: Particulate matter
RHI: Reactive hyperemia index
SD_NN_: Standard deviation of NN intervals
