# Supplementary material for: Controlled exposure to particulate matter from urban street air is associated with decreased vasodilation and heart rate variability in overweight and older adults
Source: Part Fibre Toxicol. 2015 Mar 19;12:6. doi: 10.1186/s12989-015-0081-9 (PMC4374502; doi:10.1186/s12989-015-0081-9)
Supplement: Additional file 1: — Measurement of vasomotor function. Measurement of Heart rate variability (HRV). Table S1. Blood pressure and metabolic biomarkers in subjects before and after 5-h exposure in a chamber with or without filtration of inlet air from an urban street. Table S2. Characteristics of the study participants as number or median (5;95% percentiles). [file 12989_2015_81_MOESM1_ESM.docx]

**Supplemental information**

**Controlled exposure to particulate matter from urban street air is associated with decreased vasodilation and heart rate variability in overweight and older adults**

By

Jette G Hemmingsen, Jenny Rissler, Jens Lykkesfeldt, Gerd Sallsten, Jesper Kristiansen, Peter Møller P, Steffen Loft

*Measurement of vasomotor function*

Vasomotor function was measured non-invasively using the EndoPAT2000 (Itamar Medical Ltd, Cesaria, Israel) as described previously [1,1-4]. Participants were placed comfortably in a quiet room. Their arms were placed on cushions and a blood pressure cuff was applied on one arm and the other arm served as control arm. Before each MVF measurement resting blood pressure was measured using a WelchAllyn DuraShock DS54 manometer (Welch Allyn GmbH & Co. KG, Deutschland). Finger probes were placed on the index finger on each hand. The test involved three stages: Baseline five minutes, ischemic period induced by inflating the cuff resulting in occlusion of flow through the brachial artery on the test arm for five minutes and reactive hyperemia recorded for further five minutes after cuff deflation. This flow-induced vasodilation response was stored in a computer algorithm and calculated automatically as the ratio of the post- and pre-occlusion values normalized to measurements from the control arm and reported as the Reactive Hyperemia Index (RHI). In addition, but only after each exposure scenario, we also measured the vasodilation induced by the NO donor, nitroglycerin (NTG) by the EndoPat equipment. To this end 5 mg NTG was placed under the tongue 5 min after the RHI measurement and the vasodilation response was measured on the contralateral arm for another 15 minutes or until the effect of NTG was completely subsided. The response recorded as the Nitroglycerin index (NTG-I) was calculated by the computer program as the average amplitude of the pulse wave signal after NTG divided by the average baseline pulse wave signal before NTG.

*Measurement of heart rate variability (HRV)*

HRV was measured by Actiheart (CamNtech Ltd U.K.) which is a compact, chest-worn monitoring device that digitizes the ECG signal and records the heart interbeat interval time series based on detection of the R-wawe with a time resolution of 1 ms. The Actiheart was placed on the participant’s chest before entering the exposure room and recorded for all 5 hours. For analysis of HRV two periods of 5 min were chosen: a baseline just after the participants had entered the room and an exposure dependent period for the last 5 min before leaving. During these 5 min the participants sat completely still with no activity. This was in order avoid bias in the baseline by physiological factors such as postural changes [5]. HRV analyses were conducted as previously described [6]. In brief, the raw interbeat interval time series were first subjected to a filtering procedure to exclude ectopic beats, noise, etc., and aberrant intervals were replaced with interpolated intervals. Next, the spectral components of the interbeat interval variation was extracted from 5 min periods of the filtered time series using Welch’s averaged, modified periodogram method [7]. Only time series with less than 10% interpolated beats were used for the analysis. The standard deviation (SD_NN_) of the interbeat intervals in the 5 min period was also calculated. The total power was calculated for the frequency range 0.0003-0.5 Hz, LF in the range 0.04-0.15 Hz and HF in the range 0.15-0.4 Hz. HF and LF was expressed in normalized units as HFn and LFn by dividing LF, respectively HF, with the power in the frequency range 0.04-0.5 Hz. These power ranges correspond to the Task Force recommendations [6].

**Supplement Table S1**: Blood pressure and metabolic biomarkers in subjects before and after 5-h exposure in a chamber with or without filtration of inlet air from an urban street,

Exposure Particle filtered air Non- filtered air

Before After Before After

Systolic pressure (mmHg) 120 (110;150) 130 (110;150) 130 (102.5;150) 130 (110;150)

Diastolic pressure (mmHg) 80 (65;92.5) 75 (65;97.5) 80 (67.5;97.5) 77.5 (65;90)

Total cholesterol (mmol/L) 4.5 (3.3;5.7) 4.4 (3.6;6.2) 4.6 (3.6; 6.2) 4.5 (3.4;6)

LDL cholesterol (mmol/L) 2.8 (1.6;4.1) 2.6 (1.5;4.1) 2.8 (1.8;4.3) 2.7 (1.7;4.1)

HDL cholesterol (mmol/L) 1.3 (0.9;2.3) 1.3 (0.7;2.2) 1.4 (0.8;2.2) 1.3 (0.7;2.1)

Triglycerides (mmol/L) 1 (0.5;1.7) 1.7 (0.6;3.1) 1 (0.5;1.7) 1.6 (0.7;2.8)

Glucose (mmol/l) 5.3 (4.3;6.9) 5.4 (4.1;8.2) 5.2 (4.3;6.5) 5.6 (3.7;7.7)

Hb_A1C_ (mmol/mol) 34 (28;39) 34 (26;40) 34 (26;38.5) 32 (27;40)

Hb_A1c_ haemoglobin A1c; Values are median (5^th^; 95^th^ percentiles)

**Supplement Table S2:** Characteristics of the study participants as number or median (5;95% percentiles).

Characteristics men women Total

Gender 25 35 60

Age (yr) 64 (58;80) 62 (56;77) 63.5 (57;77.5)

Height (cm) 180 (171;192) 162 (155;175) 171 (157;188)

Body mass index (kg/m^2^_)_ 28 (25;34) 28 (25;34) 28 (25;34)

**References**

1. Brauner EV, Moller P, Barregard L, Dragsted LO, Glasius M, Wahlin P, Vinzents P, Raaschou-Nielsen O, Loft S: **Exposure to ambient concentrations of particulate air pollution does not influence vascular function or inflammatory pathways in young healthy individuals.** *Part Fibre Toxicol* 2008, **5:** 13.

2. Forchhammer L, Moller P, Riddervold IS, Bonlokke J, Massling A, Sigsgaard T, Loft S: **Controlled human wood smoke exposure: oxidative stress, inflammation and microvascular function.** *Part Fibre Toxicol* 2012, **9:** 7.

3. Karottki DG, Spilak M, Frederiksen M, Gunnarsen L, Brauner EV, Kolarik B, Andersen ZJ, Sigsgaard T, Barregard L, Strandberg B, Sallsten G, Moller P, Loft S: **An indoor air filtration study in homes of elderly: cardiovascular and respiratory effects of exposure to particulate matter.** *Environ Health* 2013, **12:** 116.

4. Patvardhan EA, Heffernan KS, Ruan JM, Soffler MI, Karas RH, Kuvin JT: **Assessment of vascular endothelial function with peripheral arterial tonometry: information at your fingertips?** *Cardiol Rev* 2010, **18:** 20-28.

5. Tsuji H, Venditti FJ, Jr., Manders ES, Evans JC, Larson MG, Feldman CL, Levy D: **Reduced heart rate variability and mortality risk in an elderly cohort. The Framingham Heart Study.** *Circulation* 1994, **90:** 878-883.

6. Kristiansen J, Korshoj M, Skotte JH, Jespersen T, Sogaard K, Mortensen OS, Holtermann A: **Comparison of two systems for long-term heart rate variability monitoring in free-living conditions--a pilot study.** *Biomed Eng Online* 2011, **10:** 27.

7. Singh D, Vinod K, Saxena SC, Deepak KK: **Effects of RR segment duration on HRV spectrum estimation.** *Physiol Meas* 2004, **25:** 721-735.
